# Improved Near-Infrared Spectral Responsivity Scale

**DOI:** 10.6028/jres.105.055

**Published:** 2000-10-01

**Authors:** Ping-Shine Shaw, Thomas C. Larason, Rajeev Gupta, Steven W. Brown, Keith R. Lykke

**Affiliations:** National Institute of Standards and Technology, Gaithersburg, MD 20899-0001

**Keywords:** cryogenic radiometer, detector standards, electrical substitution, IR detector, near infrared, radiometry, responsivity

## Abstract

A cryogenic radiometer-based system was constructed at the National Institute of Standards and Technology for absolute radiometric measurements to improve detector spectral power responsivity scales in the wavelength range from 900 nm to 1800 nm. In addition to the liquid-helium-cooled cryogenic radiometer, the system consists of a 100 W quartz-tungsten-halogen lamp light source and a 1 m single-grating monochromator for wavelength selection. The system was characterized and the uncertainty in spectral power responsivity measurements evaluated. A variety of photodetectors, including indium gallium arsenide photodiodes (InGaAs), germanium (Ge) photodiodes, and pyroelectric detectors, were subsequently calibrated. Over most of the spectral range, the spectral power responsivity of the photodetectors can be measured with a combined relative standard uncertainty of 0.4 % or less. This is more than a factor of two smaller than our previous capabilities, and represents a significant improvement in the near infrared (NIR) spectral power responsivity scale maintained at NIST. We discuss the characterization of the monochromator-based system and present results of photodetector spectral power responsivity calibrations.

## 1. Introduction

Photodetectors are currently calibrated for spectral power responsivity in the visible and near-infrared (NIR) wavelength regions at the National Institute of Standards and Technology Spectral Comparator Facility (SCF) [1,2]. For the NIR spectral region (from 700 nm to 1800 nm), Ge photodiodes are used as working standards to transfer the responsivity scale to test detectors. The responsivities of the working standard detectors below 920 nm are derived by comparing their responses against those of silicon trap detectors calibrated at the NIST High Accuracy Cryogenic Radiometer facility (HACR) [3,4]. Because silicon trap detectors are stable, have uniform responsivity, good linearity, and low noise [5,6], the scale up to 920 nm can be transferred to working standard detectors with a very low relative combined standard uncertainty of approximately 0.1 %.

Prior to this work, extension of the detector responsivity scale to the NIR spectral region relied on the use of a spectrally-flat pyroelectric detector to determine the relative responsivity of NIR working standard detectors, i.e., Ge photodiodes, over the spectral range from 700 nm to 1800 nm. The spectral flatness of the pyroelectric detector was determined by measuring the spectral transmittance *τ*(*λ*) of the window material and the spectral reflectance *ρ*(*λ*) of the gold black surface material. The spectral power responsivity *S*_p_(*λ*) was then derived from

$$S_{p}\left(\lambda \right)=\tau \left(\lambda \right)\left[1-\rho \left(\lambda \right)\right]C,$$ (1)

where *C* is a calibration factor assumed to be independent of wavelength.

The inclusion of the pyroelectric detector in the chain of calibration introduced additional measurement uncertainties in addition to the uncertainties associated with the working standards. The result was a significant increase in the relative combined standard uncertainty of spectral responsivity measurements for wavelengths beyond 920 nm. At 1200 nm, the combined standard uncertainty was greater than 1 %; beyond 1400 nm, it was approximately 2.5 % [2].

One approach to reducing the uncertainties in NIR spectral responsivity calibrations is to eliminate the pyroelectric detector from the calibration chain by calibrating the working standard detectors directly against a primary standard such as the cryogenic radiometer [7,8] at HACR.

Unfortunately, it is difficult to extend the detector calibration wavelength of HACR to 1800 nm because the system is limited by a high optical power requirement – on the order of a milliwatt – that restricts HACR to the use of lasers. While a new tunable laser system is being constructed at the Spectral Irradiance and Radiance Responsivity Calibrations with Uniform Sources (SIRCUS) facility [9] that covers the NIR region, there are currently no NIR lasers available at HACR in the wavelength region of interest. Even if NIR laser sources were available, the direct calibration of working standards at HACR is very time-consuming and impractical for routine calibration.

Recently, a cryogenic radiometer compatible with low-power and divergent light from a monochromator system has been demonstrated at the National Research Council of Canada [10]. The advantages of such a system include readily available light sources, continuous spectral tunability, and the ability to calibrate detectors with the cryogenic radiometer directly, eliminating the need for transfer standard detectors such as silicon trap detectors.

We have constructed such a cryogenic radiometer-based monochromator system for use with NIST’s synchrotron ultraviolet radiation facility (SURF) for radiometric measurements in the ultraviolet [11,12]. The system was modified to enable spectral power responsivity measurements in the 900 nm to 1800 nm region. The modified system was characterized for wavelength accuracy, stray light, reproducibility, etc., and the measurement uncertainty evaluated. Using the new system, test detector responsivities can be measured with a relative combined standard uncertainty of approximately 0.4 % or less over most of the spectral range of interest. These results were used to improve the NIR spectral responsivity scale at NIST: Ge and InGaAs working standard detectors were calibrated on this facility with uncertainties in their responsivities a factor of two less than the previous NIST NIR spectral responsivity scale. In Sec. 2, we discuss the overall layout of the facility. In Sec. 3, characterizations of the monochromator system and the detectors are discussed. We present results of detector calibrations in Sec. 4, and summarize the work in Sec. 5.

## 2. Description of the Experimental Apparatus

**Fig. 1:**
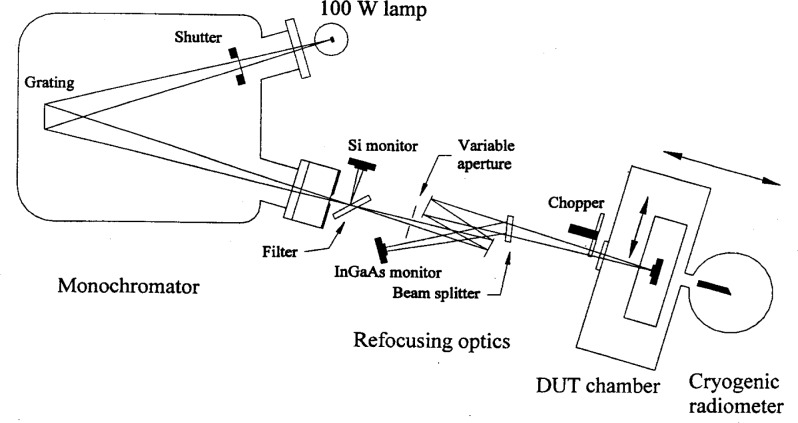
Schematic diagram of the absolute cryogenic radiometer (ACR)-based monochromator system for near infrared (NIR) radiometry.

The schematic of the experimental setup is shown in Fig. 1. A 100 W quartz-tungsten-halogen light bulb was placed at the front entrance of a 1 m, single-grating monochromator. The monochromator had a 300 lines per millimeter curved grating for light dispersion. To improve light throughput, the entrance slit was formed by the filament of the light source (≈2 mm). The exit slit was set at 2 mm, resulting in a spectral bandpass of approximately 4 nm. The beam size at the exit of the monochromator was approximately 2 mm by 2 mm. A longpass filter was installed close to the exit slit to eliminate higher-order diffracted light from the beam.

Refocusing optics, consisting of two concave mirrors, re-imaged the beam (1:1) into the detector chamber where the device under test (DUT) was placed. A fused silica vacuum window was placed at the entrance port of the detector chamber. The absolute cryogenic radiometer (ACR) was connected to the other side of the detector chamber. Both the detector chamber and the ACR were maintained in vacuum. An x-y translation stage was installed in the detector box to move the DUT in the plane perpendicular to the light path. The stage enabled the uniformity of the DUT to be mapped and it was also used to move the DUT out of the light path for measurements by the ACR. Both the detector chamber and the ACR were mounted on another single translation stage. This stage moved the whole assembly parallel to the light path to position either the DUT or the ACR onto the focal plane of the light. This ensured that the geometric measurement conditions were similar for both the ACR and the DUT. With this configuration, however, there was a difference in the distance the light travels through air between the DUT and the ACR measurements. This caused a problem in the water absorption band that will be discussed later. Additional details on the detector box and ACR operation are given in Refs. [11] and [12].

To ensure that the incident light under-filled the ACR cavity, a variable aperture was installed in front of the refocusing optics. The *f* number of the incident light can be increased by reducing the size of the aperture. During the measurement, the ratio of the signals from the ACR and the monitor was recorded while the aperture size was continuously decreased. When the incident beam under-filled the ACR cavity, the ratio was constant. An optimum aperture size was determined, ensuring that the cavity of the ACR was underfilled while the optical throughput was maximized. For typical measurements, an *f*/20 beam geometry was used.

**Fig. 2:**
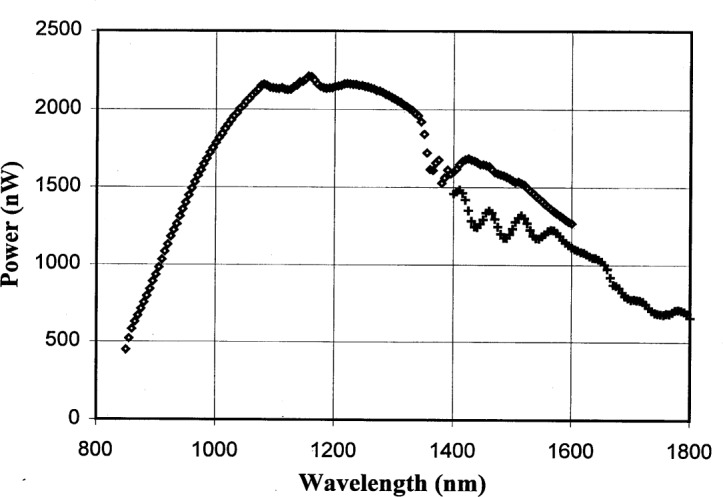
Measured optical power output from the monochromator with an exit slit size of 2 mm by 2 mm (≈4 nm resolution). Two longpass filters, with 850 nm and 1300 nm cutoff wavelengths, were used for the spectral range from 850 nm to 1600 nm (diamonds) and 1400 nm to 1800 nm (crosses), respectively.

The typical monochromator output power is shown in Fig. 2. Two different longpass order-sorting filters were used, with cutoff wavelengths of 850 nm and 1300 nm, respectively. The 850 nm filter was used for measurements in the wavelength range from 850 nm to 1600 nm and the 1300 nm filter for measurements in the range from 1400 nm to 1800 nm.

The measured signals from the ACR and the DUT are normalized by a monitor signal to eliminate the effects of drift and fluctuations from the light source. There are two monitors in the current setup: a temperature-controlled InGaAs photodiode and a silicon (Si) photodiode. The InGaAs monitor diode, located inside the mirror chamber and held at a constant temperature of 26 °C, is used to correct for source temporal intensity fluctuations. For the spectral range beyond 1600 nm, the InGaAs photodiode responsivity decreased substantially and a silicon monitor is used instead by measuring the higher-order light reflected from the order-sorting filter.

When a pyroelectric detector was calibrated, an optical chopper was installed in front of the detector. The signal from the pyroelectric detector and the reference signal from the chopper were sent to a lock-in amplifier. The output signal from the lock-in amplifier was subsequently sent to a computer for data processing.

## 3. Detector Measurement Method

The spectral responsivity of a DUT was measured by direct substitution against the ACR. In this approach, the monochromator was tuned to a particular wavelength. The ACR was then moved to the focal position and the DUT moved out of the light path. The ACR measured the absolute power of the radiation against the monitor signal at that wavelength. Finally, the DUT was moved to the focal position and the DUT response was measured and normalized by the monitor signal. The spectral responsivity *S*_x_ (SI unit A/W) of the DUT was then calculated using the following measurement equation derived similarly to the procedure described in Ref. [2]:

$$S_{x}=\frac{1}{G_{x}}\cdot \left(\frac{V_{x}-V_{d,x}}{V_{\text{mx}}-V_{d,\text{mx}}}\right)\cdot \left(\frac{V_{\text{ms}}-V_{d,\text{ms}}}{P_{\text{off}}-P_{\text{on}}}\right),$$ (2)

where *G*_x_ is the DUT transimpedance gain (in V/A); *V*_x_ and *V*_mx_ are the measured voltage from the DUT and the monitor photodiode; *V*_d,x_ and *V*_d,mx_ are the background voltage from the DUT and monitor photodiode measured with shutter closed; *P*_on_ and *P*_off_ are the electrical power (SI unit W) applied to the ACR cavity heater with the incident light and without the incident light (shutter closed); and *V*_ms_ and *V*_d,ms_ are the measured voltage and its background from monitor photodiode simultaneously recorded with the ACR measurement.

## 4. System Characterization

We can assess the contributions to the relative combined standard uncertainty of the DUT responsivity by evaluating the uncertainties of the quantities given in Eq. (1). These quantities include the measured voltage signals from the DUT and the monitor photodiode, the gain factor of the current-to-voltage converter, and the electrical power applied to the ACR cavity heater. A full treatment of the contribution of these components to the relative combined standard uncertainty in the DUT responsivity is given in Ref. [2].

Other uncertainty components that could contribute significantly to the overall uncertainty of the DUT responsivity include wavelength uncertainty, higher order diffracted light from the monochromator, and uncertainty components related to the ACR measurements. Measurements were conducted to quantify these uncertainty components.

### 4.1 Wavelength Uncertainty

**Fig. 3:**
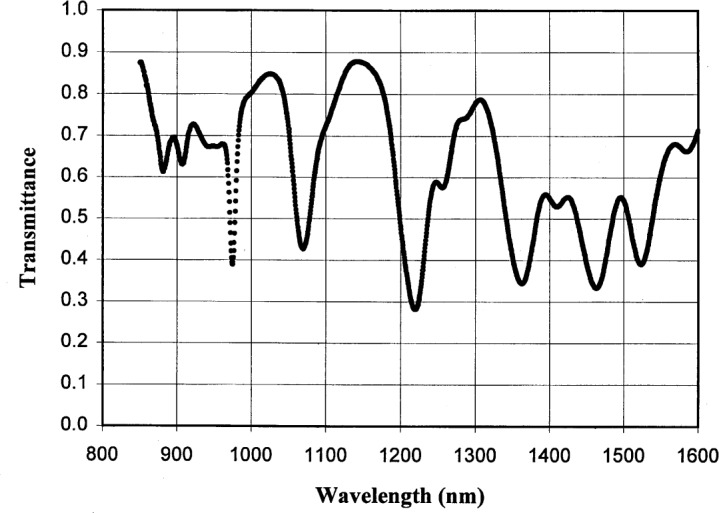
Measured transmittance of a Nd:Yb:Sm glass wavelength standard.

The wavelength uncertainty of the monochromatized light can have a significant effect on the overall calibration uncertainty in the wavelength region where there is a large variation in spectral responsivity of the device being calibrated. To assess the wavelength uncertainty of the 1 m monochromator, we measured the spectral transmittance of a Nd:Yb:Sm glass sample [13] and the result is shown in Fig. 3. The spectral transmittance of the same glass sample was subsequently measured independently by the SCF where the wavelength uncertainty is about 0.2 nm [2]. By comparing the wavelengths of the absorption peaks of the two measurements, we found the wavelength uncertainty of our system is 0.4 nm. This wavelength uncertainty is generally negligible compared to other sources of uncertainties except near the responsivity cutoff region of the InGaAs photodiodes.

### 4.2 Spectral Purity

**Fig. 4:**
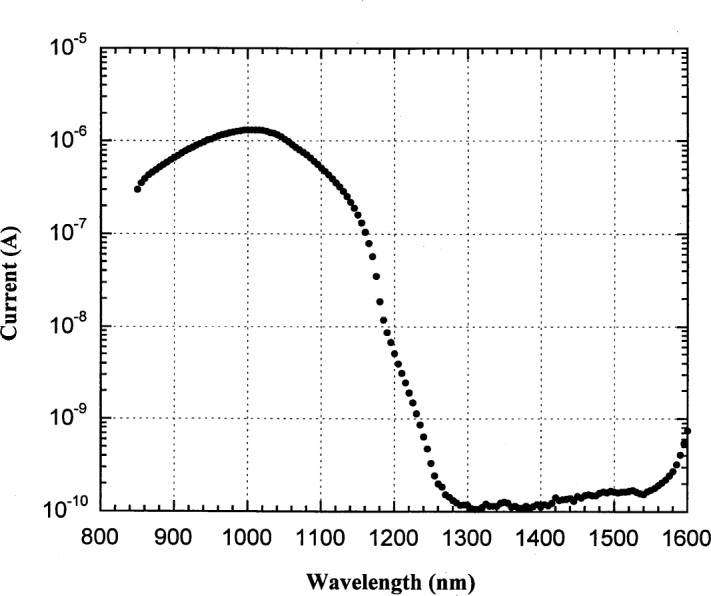
Measured response from a silicon trap detector for the study of stray light.

We performed two experiments to estimate the magnitude of spectral purity of the monochromator output in the NIR. In the first experiment, a 3-element silicon trap detector was placed in the detector chamber and the current from the trap detector was measured vs wavelength with the 850 nm longpass filter. Silicon detectors become transparent for wavelengths longer than 1200 nm and the current from the trap detector is an indication of the contribution from higher orders for wavelengths shorter than 1200 nm. As shown in Fig. 4, the contribution by higher order light is less than 0.1 % of the in-band light.

**Fig. 5:**
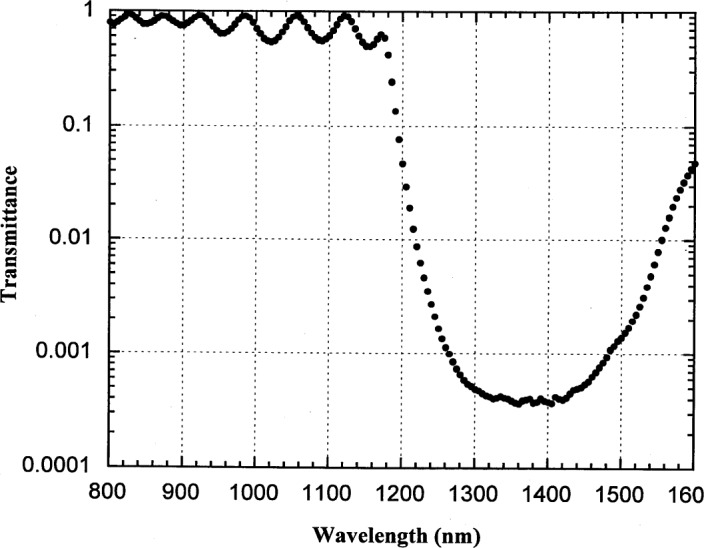
The transmittance of a laser mirror measured with the near infrared (NIR) monochromator system.

A second measurement was performed using an InGaAs photodiode as DUT and measuring the spectral transmittance of a NIR laser mirror which has very high transmittance (≈80 %) in the NIR except for a very low-transmittance (<0.1 %) in the wavelength range from 1200 nm to about 1500 nm. The absolute transmittance was measured by moving the laser mirror in and out of the beam in front of the detector box. The result is shown in Fig. 5. The transmittance of the laser window is less than 0.1 % between 1250 nm and 1500 nm. This measurement result is consistent with a separate transmittance measurement using a tunable NIR laser that shows the total stray light for the InGaAs photodiode spectral region, about 850 nm to 1700 nm, has an upper limit of 0.1 %.

### 4.3 ACR Cavity Absorption

The performance of the ACR has been previously discussed for UV measurements [12]. To extend the uncertainty analysis to the NIR, one has to check the absorptance by the chromium oxide coated receiver cavity in the NIR. We measured the total hemispherical reflectance with a flat sample coated with chromium oxide. The reflectance is about 0.1 in the NIR compared with 0.06 at 632.8 nm. The cavity absorptance at 632.8 nm was previously measured to be better than 0.9994. Because of the multiple reflections within the ACR cavity, the slight increase in the reflectivity of a single reflection in the NIR is not expected to increase the cavity absorptance uncertainty to a degree that affects the overall calibration uncertainty.

### 4.4 Intercomparison With a Si Trap Detector

Silicon trap detectors are commonly used as transfer standards in national standard laboratories [2,3]. At NIST, trap detectors are calibrated for spectral power responsivity against the HACR over the range from 406 nm to 920 nm. Our system provides reasonable optical power (more than 1 (W) for wavelengths longer than 900 nm. This gives us an overlap in spectral range from 900 nm to 920 nm to intercompare our system with the scale of a calibrated trap detector.

**Fig. 6:**
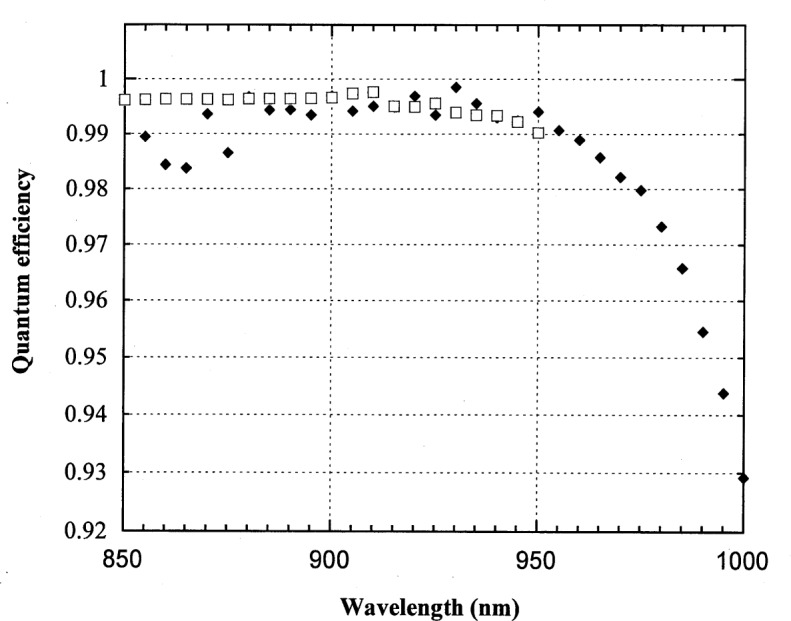
The internal quantum efficiency of a trap detector measured by the absolute cryogenic radiometer (ACR)-based setup (filled diamonds) and by the Spectral Comparator Facility (SCF) (squares).

For this intercomparison, a three-element trap detector was calibrated by the SCF and the SURF system. The results are shown in Fig. 6 with the detector response converted to external quantum efficiency. The variation between two measurements in the region from 900 nm to 920 nm is less than 0.4 %, well within the combination of the relative combined standard uncertainty of the SURF system (0.4 %) and of the SCF (0.1 %).

## 5. Detector Responsivity Calibrations

We calibrated three different types of NIR detectors with the NIR cryogenic radiometer-based monochromator system: InGaAs photodiodes, a Ge photodiode, and a pyroelectric detector. For each calibration, the responsivity uniformity of the detector active area was measured (at a typical wavelength of 1200 nm) by scanning the detector using the *x-y* translation stage in the detector box. Based on the results of the uniformity measurements, the center position of the detector was determined and used for the subsequent spectral responsivity calibration. Results of representative detector calibrations are discussed below.

### 5.1 InGaAs Photodiodes

**Fig. 7:**
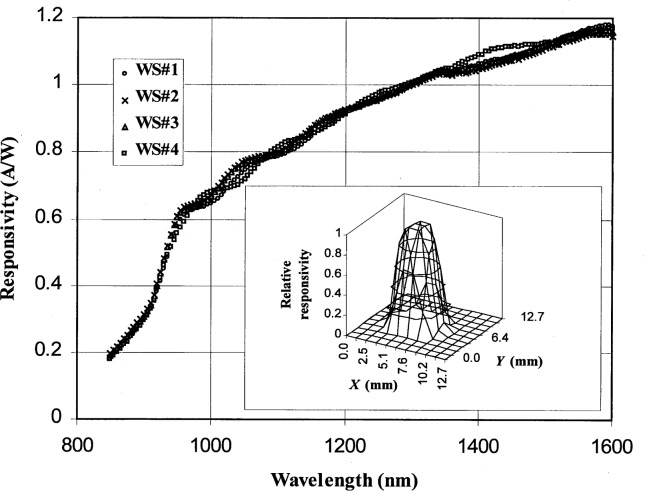
Measured spectral responsivity of four InGaAs working standards. The inset shows the spatial uniformity at 1200 nm scanned with a 2 mm by 2 mm beam.

Several InGaAs photodiodes from Ge Power Devices Inc.^1^ with an active area of 5 mm in diameter were calibrated over the spectral range from 850 nm to 1600 nm. These photodiodes were mounted onto a standard base equipped with a temperature control unit to maintain constant photodiode temperature. The temperature was set at 26 °C during calibrations. Shown in Fig. 7 are the measured responsivities of four InGaAs photodiodes. The responsivity increases slowly from 1000 nm to 1600 nm, and decreases sharply below 1000 nm and beyond 1600 nm. The cutoff at 1600 nm corresponds to the bandgap of the InGaAs active layer and the cutoff below 1000 nm corresponds to the bandgap of the topmost InP layers. A typical measurement of the responsivity uniformity with a 2 mm by 2 mm light beam at 1200 nm over the active area of the photodiode is shown in the inset of Fig. 7.

### 5.2 Ge Photodiode

**Fig. 8:**
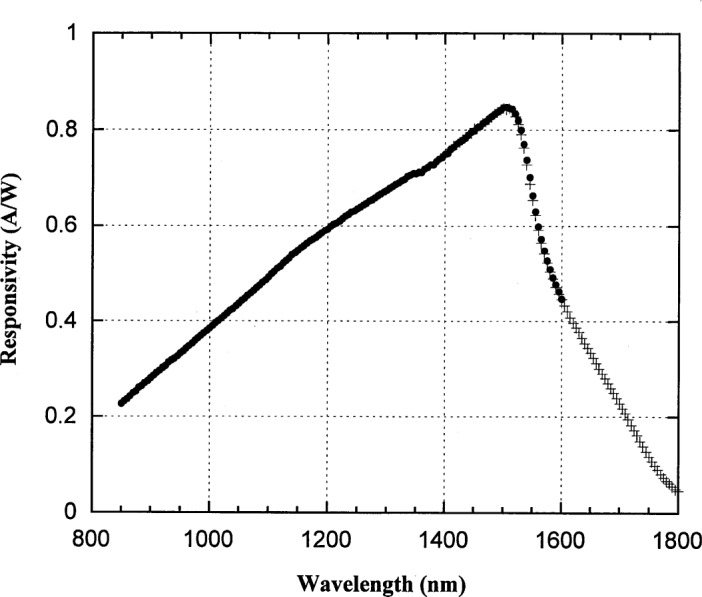
Measured spectral responsivity of a Ge working standard at −30 °C. The wavelength region from 850 nm to 1600 nm was measured with an 850 nm longpass filter (circles) and a 1300 nm longpass filter was used for measurements from 1400 nm to 1800 nm (crosses).

The spectral responsivity of a Ge photodiode was measured over the spectral range from 850 nm to 1800 nm. The temperature-controlled EG&G Judson Ge photodiode has an active area 5 mm in diameter. During calibration, the temperature was maintainted at −30 °C. The measured spectral responsivity is shown in Fig. 8. Note that two sets of measurement, with 850 nm and 1300 nm order-sorting filters, are necessary to cover the whole range.

### 5.3 Pyroelectric Detector

Pyroelectric detectors are used in the SCF as an important element in the calibration chain to extend HACR calibrations to the UV and the NIR due to their relatively flat spectral response. The spectral flatness of pyroelectric detectors contributes directly to the calibration uncertainty of the NIR scale at the SCF.

With the current setup, we were able to independently measure the NIR spectral responsivity directly against the ACR. This results in a reduction in the uncertainty of the flatness of pyroelectric detector responsivity in the NIR. It should be pointed out that the pyroelectric detector is typically used to extend the HACR calibration to the NIR therefore the relative responsivity is needed rather than the absolute spectral responsivity.

The pyroelectric detector (Oriel Corp.) has an active area 5 mm in diameter. An optical chopper modulated the light at a frequency of 10 Hz. The signal from the pyroelectric detector was directed into a lock-in amplifier (SR830 from Stanford Research Systems Inc.). A digital voltmeter (HP3457A from Hewlett Packard Inc.) digitized the analog signal from the lock-in amplifier and sent the data to a computer.

**Fig. 9:**
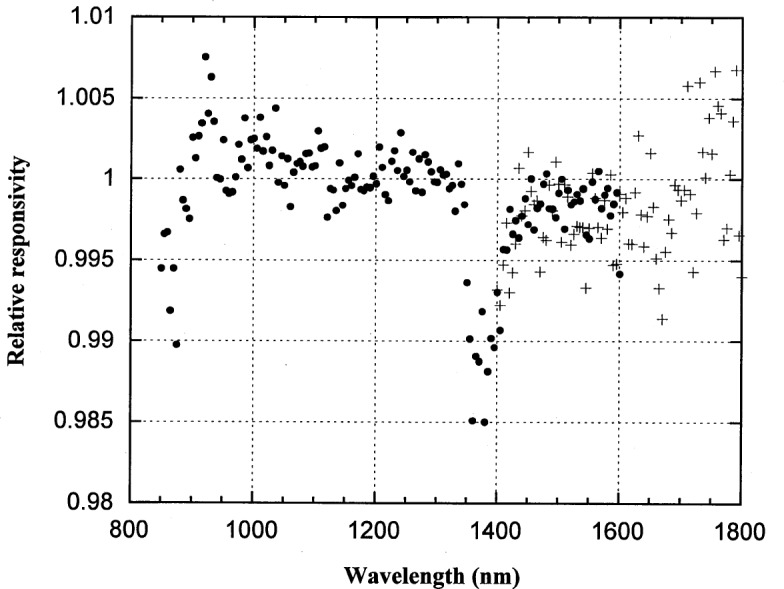
Measured relative spectral responsivity of a pyroelectric detector. The wavelength region from 850 nm to 1600 nm was measured with an 850 nm longpass filter (circles) and a 1300 nm longpass filter was used for measurements from 1400 nm to 1800 nm (crosses).

The relative spectral responsivity of the pyroelectric detector is shown in Fig. 9. The sharp dip near 1350 nm is attributed to water absorption along the optical path. As discussed previously, the optical path length is different for the ACR measurements and the pyroelectric detector measurements. This leads to a difference in the amount of light absorbed by water in the optical path. This hypothesis was confirmed by translating the pyroelectric detector near the focal position to change the length of the optical path while observing the changes of responsivity near 1350 nm. Evacuating or purging the whole optical path will be required to eliminate effects of water absorption on NIR spectral responsivity calibrations.

Outside of the water absorption region, it can be seen that the pyroelectric detector is spectrally flat to within 0.4 % for the range from about 1000 nm to 1600 nm and flat to within less than 1 % throughout the NIR region. This result can be compared with the 0.52 % uncertainty cited for the pyroelectric detector used in Ref. [2].

## 6. Measurement Uncertainty Analysis

The analysis of the uncertainty budget for the absolute responsivity measurement is similar to that of the previous UV calibration [12]. The three major sources of measurement uncertainty for the calibration procedure include ACR measurement, monitor diode measurement, and test diode measurement. For each measurement, there are Type A uncertainties (i.e., uncertainties evaluated by the statistical analysis of repeated observations [14]) and Type B uncertainties (i.e., uncertainties calculated by other means) caused by systematic effects (wavelength uncertainty, spectral purity, detector spatial response uniformity, etc.). For the ACR optical power measurement, the dominant source of uncertainty is the type A uncertainty caused by the noise floor of the ACR (≈2 nW). With the typical measured light power of 1 µW, the noise floor contributes 0.2 % of uncertainty. By including other uncertainty components for cavity absorption, scale factor, and nonequivalence [12], the combined relative standard uncertainty of the ACR power measurement increases to 0.22 %.

Listed in Table 1 are the assigned uncertainty components of the InGaAs and Ge spectral responsivity measurements for wavelengths between 950 nm to 1600 nm. The combined relative standard uncertainty is 0.38 %. For 1600 nm to 1800 nm, the combined relative standard uncertainty increases to about 1 % because of lower light power from the monochromator and therefore larger uncertainty from the ACR power measurement.

### 6.1 InGaAs

**Fig. 10:**
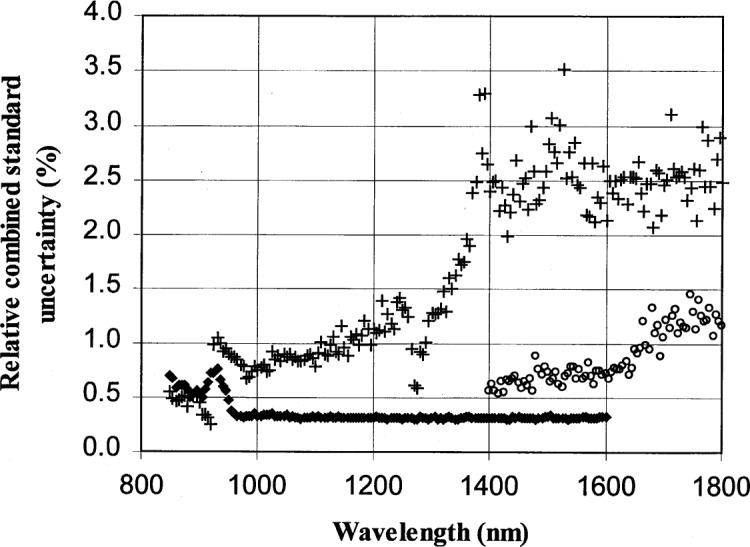
The relative combined standard uncertainty for InGaAs photodiode calibration. The wavelength region from 850 nm to 1600 nm was measured with an 850 nm longpass filter (diamonds) and a 1300 nm longpass filter was used for measurements from 1400 nm to 1800 nm (circles). Also shown is the Spectral Comparator Facility (SCF) uncertainty prior to this work (crosses).

A more detailed calculation of the relative combined standard uncertainty in the absolute responsivity of an InGaAs photodiode based on the measured type A uncertainty of the test diode, monitor diode, and ACR measurement is shown in Fig. 10. Also shown in Fig. 10 is the uncertainty derived from the SCF. There is more than a factor of 2 reduction of calibration uncertainty. The reduction in uncertainty is even more pronounced in the region from 1400 nm to 1600 nm.

**Fig. 11:**
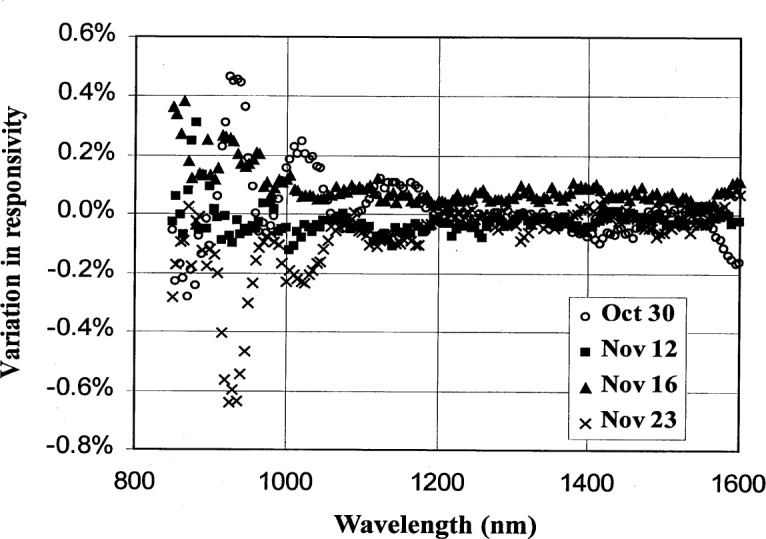
Variation of the responsivity of an InGaAs photodiode measured during a four-week period from October to November 1998.

The reproducibility of the InGaAs photodiode calibration was also evaluated. The detector spectral responsivity was measured a total of four times over a period of a month. As shown in Fig. 11, the reproducibility of the measurements was better than 0.2 % from 1000 nm to 1600 nm, and with increased variation in the spectral cutoff region from 850 nm to 1000 nm.

With lowered uncertainty, the InGaAs photodiodes calibrated by this work are currently used as working standards for NIR detector calibration.

**Fig. 12:**
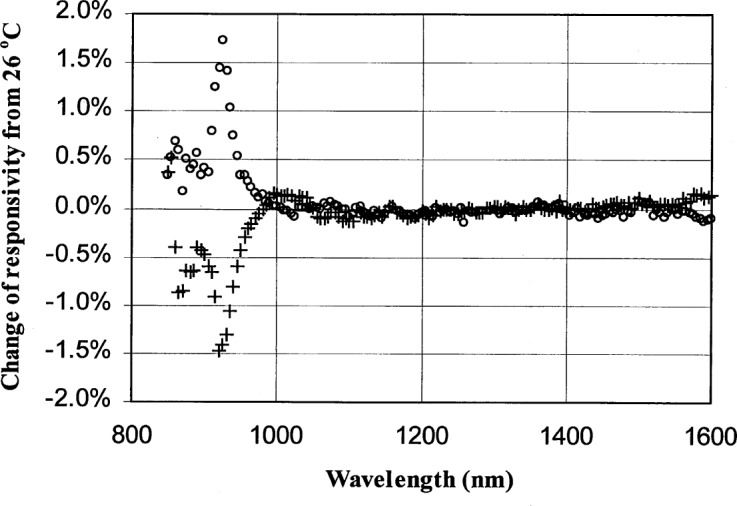
Variation of the responsivity of an InGaAs photodiode at 21 °C (circles) and 31 °C (crosses) compared to its responsivity at 26 °C.

Finally, it is of interest to study the InGaAs photodiode cutoff region below 1000 nm. The response of the detector near these cutoff regions is very sensitive to a number of factors, such as wavelength and temperature variation. Consequently, these effects contribute to the increase in uncertainty as shown in Fig. 10. We measured the temperature dependence of the InGaAs photodiode response by measuring its responsivity at temperatures 5 °C above and below the normally operated temperature of 26 °C. As shown in Fig. 12, there is a negligible change in detector responsivity except for the cutoff region, where a temperature-dependent responsivity as large as 0.3 %/°C was observed.

### 6.2 Ge

**Fig. 13:**
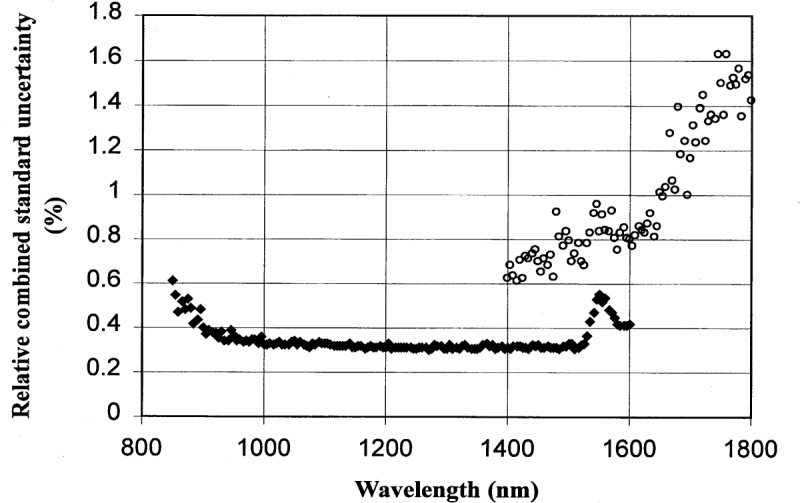
The relative combined standard uncertainty for Ge photodiode calibration. The wavelength region from 850 nm to 1600 nm was measured with an 850 nm longpass filter (filled diamonds) and a 1300 nm longpass filter was used for measurements from 1400 nm to 1800 nm (circles)

The combined standard uncertainty of Ge photodiode calibration can be derived in a manner similar to the evaluation of InGaAs photodiode calibration uncertainty. The result is shown in Fig. 13. The combined standard uncertainty is very close to that of the InGaAs photodiodes except near the wavelength of 1550 nm where the fast decrease of the Ge photodiode responsivity caused the wavelength uncertainty to dominate over other sources of uncertainty. In this region, InGaAs photodiodes are better suited as calibration working standards.

## 7. Conclusions

We extended a cryogenic radiometer-based monochromator system, developed for UV detector radiant power responsivity measurements, into the NIR spectral region to directly calibrate a variety of detectors—including InGaAs, Ge, and pyroelectric detectors—in the wavelength region from 900 nm to 1800 nm. InGaAs photodiodes were calibrated for spectral responsivity over the range from 1000 nm to 1600 nm with a relative combined standard uncertainty of less than 0.4 % and less than 1.5 % throughout the entire 900 nm to 1800 nm region. This is more than a factor of two reduction in uncertainty over the original NIR spectral responsivity scale of NIST. As a result of this work, InGaAs photodiodes calibrated directly against the current cryogenic radiometer system are now used as NIST’s NIR working standards.

The reduction in uncertainty over the existing NIR scale is largely due to calibrating the detectors directly against a cryogenic radiometer, thereby eliminating the pyroelectric detector from the calibration chain. Based on this technique, further reduction of the uncertainty in NIR spectral responsivity measurements is possible by using higher power monochromatized light, such as that produced by a laser. With the development of these sources, we expect to achieve further reduction in NIR responsivity measurement uncertainty.

## Figures and Tables

**Table 1:** Estimated components of the combined relative standard uncertainty of measured InGaAs spectral responsivity from 950 nm to 1600 nm. The 1 µW optical power is the typical power in this region

Source of uncertainty	Component of uncertainty (%)
ACR power measurement with 1 µW optical power	0.22
Monitor diode current measurement	0.10
Test diode current measurement	0.04
Wavelength scale and spectral bandwidth	0.15
Higher order and out-of-band stray light	0.10
Test diode positioning	0.20
Alignment	0.10
Relative combined standard uncertainty	0.38

## Footnotes

1 Certain commercial equipment, instruments, or materials are identified in this paper to foster understanding. Such identification does not imply recommendation or endorsement by the National Institute of Standards and Technology, nor does it imply that the materials or equipment identified are necessarily the best available for the purpose.
